# Psychotherapeutic benefits of compassion-focused therapy: an early systematic review

**DOI:** 10.1017/S0033291714002141

**Published:** 2014-09-12

**Authors:** J. Leaviss, L. Uttley

**Affiliations:** School of Health and Related Research (ScHARR), University of Sheffield, UK

**Keywords:** Compassion, compassionate mind, compassion-focused therapy, mindfulness, psychological health, psychotherapy

## Abstract

**Background.:**

Compassion-focused therapy (CFT) is a relatively novel form of psychotherapy that was developed for people who have mental health problems primarily linked to high shame and self-criticism. The aim of this early systematic review was to draw together the current research evidence of the effectiveness of CFT as a psychotherapeutic intervention, and to provide recommendations that may inform the development of further trials.

**Method.:**

A comprehensive search of electronic databases was undertaken to systematically identify literature relating to the effectiveness of CFT as a psychotherapeutic intervention. Reference lists of key journals were hand searched and contact with experts in the field was made to identify unpublished data.

**Results.:**

Fourteen studies were included in the review, including three randomized controlled studies. The findings from the included studies were, in the most part, favourable to CFT, and in particular seemed to be effective for people who were high in self-criticism.

**Conclusions.:**

CFT shows promise as an intervention for mood disorders, particularly those high in self-criticism. However, more large-scale, high-quality trials are needed before it can be considered evidence-based practice. The review highlights issues from the current evidence that may be used to inform such trials.

## Introduction

### The benefits of an early systematic review of compassion-focused therapy (CFT)

This review examines the psychotherapeutic effects of CFT, a relatively novel form of psychotherapy. CFT has received increasing interest as an intervention for a range of psychological disorders, including depression, anxiety and schizophrenia. The relevance of evidence-based medicine (EBM) to clinical psychology is increasingly recognized (Spring & Neville, 2011). In this review we aimed to determine whether, based on the current research evidence, CFT represents a plausible choice of treatment in evidence-based practice.

As CFT is a relatively new emerging therapy, the number of well-designed prospectively registered randomized controlled trials (RCTs) may currently be limited. However, the increased interest in compassion, as evidenced by numerous papers (e.g. Neff *et al.* 2007; Barnard & Curry, 2011), suggests a need for the current state of evidence to be reviewed to demonstrate whether the increasing popularity of CFT is supported by a sound research base. This early systematic review of the current evidence includes all study designs with a view to obtaining important information on the acceptability and tolerability of CFT to patients. No systematic appraisal of the evidence for CFT has been undertaken to date.

### Origins of CFT

CFT uses a definition of compassion grounded in Buddhist tradition, which defines compassion as ‘a sensitivity to suffering in self and others, with a commitment to try to alleviate and prevent it’ (The Dalai Lama, 2001). CFT is part of a growing global movement that recognizes the potential of compassion to provide benefits in a range of sectors, from business, education and healthcare to science, research and the environment (Charter for Compassion; http://charterforcompassion.org/). CFT was founded by Paul Gilbert (2000) in response to the observation that many people, in particular those high in shame and self-criticism, were experiencing difficulties generating kind and self-supporting inner voices when engaging in traditional therapy. It was observed that although these individuals were able to engage with cognitive and behavioural tasks, they still responded poorly to therapy (Rector *et al*. 2000; Bulmarsh *et al.* 2009). CFT was developed initially to help those individuals create affiliative feelings towards themselves, and to help them develop a more compassionate inner voice. CFT was based upon a growing body of neuroscientific evidence that demonstrated that affiliative motives and emotions can have a major impact on self and affect regulation (Cozolino, 2002; Depue & Morrone-Strupinsky, 2005). This research explores the interaction between three human affect regulation systems: threat protection, seeking and acquiring, and soothing. Gilbert (2014) proposes a framework of action for the biological mechanisms underpinning compassion that is based on the principles of evolutionary biology. A comprehensive overview of the theory and processes underpinning CFT is presented in Gilbert (2014); a brief summary of salient issues is presented below.

Gilbert conceptualizes compassion from an evolutionary perspective, focusing on the evolution of the mammalian affiliative system. The basis of this model lies in the neuroscience behind these affect systems. Basic motivational systems have evolved in humans and other mammals that enable us to seek out resources and avoid harm. These systems are responsible for a range of corresponding emotions, including competing and social ranking, cooperation/sharing, caring and nurturing, and seeking and responding to care. Some of the motivational systems that are associated with interpersonal relating have been implicated in psychopathology (Nesse, 2005; Buss, 2009). (i) The threat and protection system is central to the ability to detect and respond to threat (LeDoux, 1998). Activation of this system can give rise to attention focusing/bias, and results in negative emotions such as anger, anxiety and disgust. These emotions lead to fight, flight or submission behaviours. (ii) The system associated with seeking and acquiring has a motivational function, directing attention towards rewards and resources (e.g. food, sexual opportunities), and gives rise to the positive emotions of drive, excitement and vitality. (iii) The contentment/soothing system evolved alongside attachment/affiliation (Depue & Morrone-Strupinsky, 2005). Although this system also gives rise to positive emotions, these are different to those produced by the drive system, and include peacefulness, well-being, not-seeking and contentment, or ‘rest and digest’. Social bonding and soothing behaviours have been shown to mitigate the destructive effects of negative environmental events (Coan *et al.* 2006). The hormone oxytocin has been shown to play a key role in the creation and organization of affiliative behaviour (e.g. Kirsch *et al.* 2005).

CFT aims to redress imbalances within these three affect regulation systems, seeking to help individuals who have difficulty accessing the soothing system in response to threat. This difficulty may have an environmental or a biological basis (Belsky & Beaver, 2011), for example understimulation of the soothing system in early life (Gilbert, 2014). CFT aims to help such individuals respond to self-criticism with self-kindness and compassion, with the goal of treatment being improved psychological well-being. A key part of this process is to help the individual understand that many cognitive biases/distortions are built-in biological processes, constructed by genetics and the environment. CFT encourages individuals to develop compassion motivation and practise compassionate behaviours to access the soothing systems.

### Compassion and psychopathology

Interpersonal relating is central to the affect regulation systems, and compassion is proposed to emerge from these evolved social motivational systems. Although there are several models of compassion (see MacBeth & Gumley, 2012), they all propose a negative relationship between compassion and psychopathology. The meta-analysis by Macbeth & Gumley (2012) supports this relationship. They report a large effect size for the relationship between compassion and psychopathology (depression, anxiety and stress), with high levels of compassion associated with lower levels of psychopathology.

CFT recognizes that compassion flows in three directions: compassion we can feel for another or others, compassion we can feel from others to ourselves, and compassion we can direct towards ourselves (self-compassion) (Gilbert, 2014). CFT describes the ‘underpinning theory and process of applying a compassion model to therapy’ (Gilbert, 2009*a*) whereas compassionate mind training (CMT) describes ‘specific activities designed to develop compassionate attributes and skills’ (Gilbert, 2009*b*).

### Evidence of compassion and psychological well-being

Compassion regulates negative affect through caring behaviours and expressing and communicating feelings of warmth and safeness. Several studies have explored the relationships between self-compassion and well-being. Survey research using scales of self-compassion show self-compassion to be correlated with symptom severity and quality of life (Van Dam *et al.* 2011), well-being (Neff *et al*. 2007; Neely *et al.* 2009) and maternal support and family functioning (Neff & McGehee, 2010). Increases in self-compassion have been found to correlate with a decrease in psychiatric symptoms, interpersonal problems and personality pathology (Schanche *et al.* 2011). Although such evidence cannot prove direct cause, it does indicate that the ability to show self-compassion is correlated with psychological well-being, with some research suggesting that self-compassion may be a better predictor of anxiety and depression than mindfulness (Van Dam *et al.* 2011).

### Compassion as a psychotherapeutic intervention

The application of compassion as a psychotherapeutic intervention has received increasing attention. For the therapist, using a compassion-based therapy involves enabling individuals to develop self-compassion, compassion to others, and openness to compassion from others, in particular in response to adversity or threatening situations. Jazaieri *et al.* (2013) demonstrated that a programme of compassion cultivation training (CCT) was successful at enhancing compassion, and showed therefore that compassion can be taught and learnt through training. This study is notable in that it showed that fear of compassion can be reduced. Compassion-based meditation (CM) and loving kindness meditation (LKM) have been shown to reduce stress (Lutz *et al.* 2008) and to increase sensitivity to distress in others. Similarly, the practice of ‘loving kindness’-based meditation has been shown to increase positive emotions, mindfulness, feelings of purpose in life and social support, and to decrease illness symptoms (Fredrickson *et al.* 2008). From a biological perspective, the practice of CM has been shown to reduce stress-linked immune responses (Pace *et al.* 2009). Practising compassion-based exercises such as letter-writing to oneself has been shown to reduce symptoms of depression (Leary *et al.* 2007) and promote coping strategies (Neff & Vonk, 2009). A review of the literature on LKM and CM (Hofmann *et al.* 2011) suggests that both are associated with an increase in positive affect and a decrease in negative affect, and that they may enhance activation of the brain areas involved in emotional processing and empathy; and neuroendocrine studies suggest that CM reduces stress-induced distress and immune response. In addition, Hofmann *et al.* (2011) found that elements of LKM and CM can be trained within a relatively short period of time.

### Core principles of CFT

CMT is at the core of CFT. CMT aims to help clients to learn the key skills required to develop the key aspects and attributes of compassion, cited as care for well-being, sensitivity, distress tolerance, empathy and non-judgement (Gilbert, 2009*b*). Specific skills needed to achieve these attributes are multi-modal and common to other psychotherapies. These skills include compassionate reasoning, compassionate behaviour, compassionate imagery, compassionate feeling and compassionate sensation (Gilbert, 2009*b*). Some of the key steps involved in sessions of CMT are the use of imagery, compassionate thinking to the self and others, responding to self-criticism through self-compassion and practising compassionate behaviour, often complimented with letter or diary writing. CFT encourages the client to focus on, understand and feel compassion to the self during negative thought processes, with a strong focus on nurturing compassion within the self. Gilbert (2009*b*) argues that CFT may be used as a framework within which to focus other psychological interventions, as these may become more effective once the affiliative system has been stimulated. Individuals with a highly self-critical ‘inner voice’ may struggle with other evidence-based therapies, so helping these individuals to develop a more compassionate, encouraging ‘inner voice’ may enable better engagement. CFT is therefore proposed for use as a multi-modal therapy, based on a scientist–practitioner model rather than belonging to a single ‘school of therapy’.

### Aims of the current review

This review aimed to draw together the current research evidence of the effectiveness of CFT as a psychotherapeutic intervention, and to make recommendations for future trials based on the findings. As CFT is a relatively new intervention, search criteria were kept wide to include both clinical and non-clinical populations.

## Method

Inclusion criteria for studies were as follows: (i) *Population:* participants with clinical diagnosis of any psychological disorder or self-reported symptoms of any psychological disorder. As the intervention is a relatively new form of psychotherapy, studies of participants without clinical psychological diagnoses were also included. (ii) *Intervention:* studies assessing the effectiveness of CFT as a psychotherapeutic intervention were included. Studies of CFT delivered by a clinician were included, along with studies of self-help exercises designed to promote self-compassion but without the support of a clinician (see Table 1 for descriptions of included interventions). Correlational studies of self-compassion and psychological outcomes were excluded. Studies of non-compassion-based mindfulness interventions were excluded, as were studies of LKM that were not explicitly compassion focused. (iii) *Comparators:* all comparators including any other psychotherapy, any psychopharmacological interventions, no treatment, and treatment as usual (TAU). (iv) *Outcomes:* primary outcomes to be considered in the review were psychotherapeutic outcomes: improvement in psychological symptoms; level of self-compassion or self-criticism; interpersonal and social functioning; quality of life; and use of prescribed medicine. Secondary outcomes of interest were: biological, neurophysiological or immunological changes. (v) *Study types:* RCTs were included in the assessment. Data from non-randomized studies, case series and observational studies were considered for inclusion as evidence if data available from RCTs were limited. Systematic reviews were included if they provided additional data meeting the inclusion criteria. Other exclusion criteria were: studies based on animal models; editorials; opinion pieces; reports published as meeting abstracts only where insufficient details were reported to allow inclusion; book chapters; studies that did not present data for the included outcomes.

**Table 1.** Summary description of included studies

Study (country)	*n*	Design	Population	Intervention	Control	Number of sessions	Measure	Main outcomes
Braehler *et al*. 2013 (UK)	40	RCT	Schizophrenia-spectrum disorder with psychotic features	Group CFT for psychosis + TAU. Focused on reduction of shame, stigma, self-blame, development of compassion. Mindfulness, appreciation, compassion imagery and attention and reflection through writing	TAU. Free to vary post-randomization but could include: psychotropic medication, occupational therapy, day centre support or psychological therapies	16 weekly 2-h sessions	NRSS, BDI, PANAS, FORSE, PBIQ-R, CGI-I	CFT showed significantly more clinical global improvement and more compassion in narratives than TAU. Increase in compassion in CFT group associated with decrease in BDI depression, PBIQ shame, entrapment and social marginalization, FORSE intrusiveness and fear of relapse
Kelly *et al.* 2010 (Canada)	119	RCT	Smokers	Self-compassion intervention based on CMT. Imagery-based self-talk exercises, designed to stimulate soothing-affiliation system	1 × baseline control involving daily self-monitoring exercises. 2 × experimental control (self-energizing and self-controlling imagery exercises)	Daily exercises for 3 weeks. Two laboratory sessions 3 weeks apart, baseline control 20 min + experimental imagery 25 min	Cigarettes smoked per day, SSC-SF, Trait Self-criticism Scale of the DEQ, Imagery Vividness Rating Scale, SeCS	No differences in rate of smoking reduction between three experimental imagery groups but all reduced smoking more than baseline control. Effects moderated by trait self-criticism, readiness to change and vividness of imagery
Shapira & Mongrain, 2010 (Canada)	1002	RCT	Non-clinical sample recruited through the internet	Self-compassion intervention. Letter-writing exercises about distressing event and providing compassion to themselves	1 × experimental control: optimistic thinking intervention, letter-writing visualizing future with resolved issues. 1 × control condition wrote freely about early memory	Daily exercises over 7-day period	DEQ, CES-D, SHI	Both experimental interventions resulted in significant increases in happiness at 6 months and significant decreases in depression at 3 months. Effects moderated by self-criticism and levels of dependence
Beaumont *et al*. 2012 (UK)	32	Non-RCT	Requiring treatment after trauma	CMT. Loving, caring, accepting imagery, compassionate letter-writing, grounding work + CBT (as control)	CBT techniques including cognitive restructuring, behavioural activation, graded exposure, relapse prevention and Socratic dialogue	Twelve weekly sessions (duration n.r.)	HADS Anxiety and Depression, IES Avoidance Hyperarousal Intrusion, SeCS Compassion	Significant reduction in depression, avoidance in both conditions. Improvement in scores greater for combined CMT + CBT. No difference in anxiety levels between groups. Greater improvement in self-compassion for CMT + CBT than CBT
Kelly *et al.* 2009 (Canada)	75	Non-RCT	Distressed chronic acne sufferers	CFT. Self-soothing condition. Slideshow about compassionate self-talk, visualization of compassionate image, compassionate letter-writing. Focus on warmth, acceptance, reassurance, desire to soothe distress	Attack-resisting intervention. Visualize confident, resistant, resilient image. Focus on strength, logic perseverance, self-confidence. Control condition: no exercises	Two weekly sessions. One × 1 h, one × n.r. + three × per day exercises for 2 weeks	DEQ, BDI, ESS, SKINDEX-16	Depression reduced across conditions. Only attack-resisting condition lowered depression more than control. Moderating effect of self-criticism. Both experimental conditions showed greater reduction in shame than control
Gilbert & Irons, 2004 (UK)	8	Observational	Individuals from a self-help depression group who regard themselves as self-critical	Compassionate image use. Diaries recording critical thinking	None	Four 1-h sessions over 7 weeks (three consecutive weekly meetings + 4-week follow-up)	HADS, diary and quantitative ratings for self-criticism and self-soothing	No significant change in self-criticism. Significant increase in self-soothing/compassion, ease of generating images in a self-critical situation
Gilbert & Procter, 2006 (UK)	6	Observational	Patients currently in treatment for major/severe long-term complex mental health difficulties	CMT. Group therapy exploring self-criticism, compassion, self-attacking	None	Twelve weekly 2-h sessions	HADS, FSCS, FSCRS, Social rank variables, OAS, Social Comparison Scale, Submissive Behaviour Scale, weekly diary self-attacking/self-soothing	Significant reduction in HADS anxiety and depression, diary self-criticism, shame, inferiority and submissive behaviour.. Significant increases in self-compassion, reassure-self
Laithwaite *et al*. 2009 (UK)	18	Observational	Individuals with primary diagnosis of schizophrenia or bipolar affective disorder	Three modules of CFT-based programme. Understanding psychosis and recovery, understanding compassion and developing the ideal friend, developing plans for recovery after psychosis	None	Twenty sessions over 10 weeks	BDI-II, SeCS, SCS, RSE, SIP-AD, OAS	Significant improvement on BDI, SCS, OAS, RSE. No change on Self-Compassion Scale or SIP-AD
Lucre & Corten, 2013	10	Observational	Individuals with chronic personality disorder and who regard themselves as self-critical	CFT groupwork programme. Exercises to develop capacity for self-soothing. Compassion-focused imagery exercises	None	Sixteen weeks with 1-year follow-up	SCS, SBS, OAS, FSCRS, DASS21, CORE	Significant decrease in depression and stress. Reduction in feelings of shame and social comparison. Reduction in self-hatred. Significant increase in self-reassurance
McEwan & Gilbert, unpublished data (UK)	45	Observational	Non-clinical volunteers	Four audio recordings including, soothing rhythm breathing, compassionate self- imagery, compassionate other imagery. Participants chose which exercise to practise	None	At least 5 min a day over 2 weeks. Amount of time varied by individual from several times a day to every other day	Social Safeness and Pleasure Scale, SeCS, Fears of Compassion Scale, Types of Positive Affect Scale, Experiences in Close Relationships, FSCSR, DASS	Significant increases in self-compassion, social safeness, active and relaxed positive affect and self-reassurance. Significant reductions in self-coldness, fear of comparison for others, avoidance of close relationships, inadequate self-criticism, depression, anxiety and stress
Judge *et al.* 2012 (UK)	42	Observational	Range of diagnoses including depression, anxiety, OCD, bipolar, personality disorders, social anxiety, deliberate self-harm. Those referred on to CFT were high in self-criticism and shame	Group CFT	None	Between 12 and 14 weekly sessions of 2 h with a 15-min break	Depression, anxiety, inadequate self, hated self, reassured self, self-correction, self-persecution, internal shame, external shame, social comparison, submissive behaviour, self-critical thought, self-soothing thoughts (BDI, BAI, FSCRS, FSCS, ISS, OAS, SCS, SBS, weekly diary for self-attacking/self-soothing)	Significant improvement for all measures apart from self-correction
Gale *et al.* 2014 (UK)	139	Observational	Individuals diagnosed and being treated for an eating disorder	Group-based CBT, integrating CFT. Two-step treatment programme consisting of didactic teaching with in-session written activities and homework, designed to increase understanding of their eating disorder and be actively involved in deciding if they are ready to engage in treatment	None	Step 1: patients are offered a 4-week 2-h per week group-based psycho-education programme, followed by a 20-session group-based recovery programme, taking place over 16 weeks, with two sessions a week for the first 4 weeks and then 12 weekly sessions. Groups last 2–2.5 h, with 2 h of homework each week	EDE-Q; SEDS; CORE-OM	EDE-Q: significant improvement in all EDE-Q subscales after intervention. People with bulimia nervosa improve most between times 1 and 5. SEDS: significant improvement in all subscales except low assertiveness. People with bulimia nervosa improved more than the other two groups between times 1 and 5. CORE-OM: significant improvement for all subscales after intervention. People with bulimia nervosa improved the most between time 1 and 5
Mayhew & Gilbert, 2008 (UK)	3	Case series	Individuals diagnosed with schizophrenia experiencing auditory hallucinations	CMT on a one-to-one basis. Focus on understanding and compassionate to safety behaviours. Discussion about self-compassion, tasks that can be used to develop self-compassion	None	Twelve weekly 1-h sessions	BAVQ, FSCS, FSCRS, SCL-90, Voice Rank Scale, SeCS, weekly diary	All participants showed decrease in SCL-90, inadequate-self scores. All showed improved BAVQ (total scores reduced, all voices became less malevolent and less persecuting). Two of three heard more reassuring voices
Ashworth *et al.* 2011 (UK)	1	Case study	A 23-year-old female with acquired brain injury after road traffic accident	CFT and CBT sessions targeting low self-esteem, self-criticism, CMT to self-soothe using and brainstorming self-nurturer imagery	None	Twenty-four weekly 1-h and 50-min sessions	Robson SCQ, BAI, BDI, State Trait Anger Expression Inventory, eating disorder symptoms	Reliable decreases in BAI, BDI and SCQ. Anger Expression Inwards Scale fell within normal range. Beliefs relating to key cognitions indicated improvement

CFT, Compassion-focused therapy; CMT, compassionate mind training; CBT, cognitive behavioural therapy; ISS, Injury Severity Scale; SCS, Social Comparison Scale; FSCS, Functions of the Self-Criticizing/Attacking Scale; FSCRS, Forms of the Self-Criticizing/Attacking and Self-Reassuring Scale; DASS, Depression Anxiety and Stress Scale; SBS, Submissive Behaviour Scale; OAS, Other As Shamer Scale; EDE-Q, Eating Disorder Examination Questionnaire; SEDS, Stirling Eating Disorders Questionnaire; CORE-OM, Clinical Outcomes in Routine Evaluation – Outcome Measure; SIP-AD, Self-Image Profile for Adults; RSE, Rosenburg Self-Esteem measure; SeCS, Self-Compassion Scale; HADS, Hospital Anxiety and Depression Scale; DEQ, Depressive Experiences Questionnaire; BDI, Beck Depression Inventory; BAI, Beck Anxiety Inventory; SCQ, Self-Concept Questionnaire; ESS, Experiences of Shame Scale; IES, Impact of Events Scale; CES-D, Centre for Epidemiological Studies Depression Scale; SHI, Steen Happiness Index; SSC-SF, Smoking Stage of Change – Short Form; PANAS, Positive and Negative Affect Scale; FORSE, Fear of Recurrence Scale; PBIQ-R, Personal Beliefs about Illness Questionnaire – Revised; CGI-I, Clinical Global Impression – Improvement Scale; NRSS, Narrative Recovery Style Scale; TAU, treatment as usual; BAVQ, Belief About Voices Questionnaire; n.r., not recorded; RCT, randomized controlled trial; SCL-90, Symptom Checklist-90; OCD, obsessive–compulsive disorder.

A comprehensive search was undertaken to systematically identify literature relating to the effectiveness of CFT as a psychotherapeutic intervention. The search strategy comprised the following main elements: (1) searching of electronic databases; (2) contact with experts in the field; and (3) scanning bibliographies of retrieved papers. The following electronic databases were searched from inception for published trials and systematic reviews: MEDLINE: Ovid; MEDLINE In-Process and Other Non-Indexed Citations: Ovid; EMBASE: Ovid; PsychINFO: OvidSP; Web of Science. Additional searches were conducted for unpublished (ongoing or completed) studies in Bandolier, Clinical Trials.gov, and Current Controlled Trials, Cochrane Central Register of Controlled Trials. No date or language restrictions were applied. To avoid missing relevant studies, terms used for searching the electronic databases did not specify target outcomes for the intervention, and instead focused on the intervention itself (i.e. CFT). Terms used for the searches were as follows: (treatment.tw OR therapy.tw OR training.tw OR therap$.tw OR intervention.tw) AND (compassion.tw OR compassionate.tw OR compassionate-mind.tw). Literature searches were conducted during April 2012. References were collected in a bibliographic management database, and duplicates removed.

Study selection was conducted by two reviewers. In the first instance, titles and abstracts were examined for inclusion. The full manuscripts of citations judged to be potentially relevant were retrieved and further assessed for inclusion. Discrepancies between reviewers’ decisions were discussed, and if no agreement could be reached, were resolved by referring to the review's clinical expert. Data were extracted without blinding either to authors or journal. Data were extracted by one reviewer and checked by a second reviewer. Where multiple publications of the same study were identified, quality and data extraction were based on all relevant publications, and listed as a single study.

### Quality assessment

Because of a lack of RCTs, this review drew mainly on non-randomized research evidence. To assess the risk of bias of both the randomized and non-randomized studies, we focused on the following core quality domains: selection/allocation of participants; blinding (where relevant); compliance/fidelity; and reporting of findings. We used a modified version of the Cochrane risk of bias tool (Higgins *et al.* 2011) to assess these criteria. Quality assessments were conducted by one reviewer and checked by another. Discrepancies were resolved through discussion between the two reviewers.

### Data synthesis methods

The prespecified outcomes were tabulated and discussed within a descriptive synthesis. Insufficient and heterogeneous data meant that statistical synthesis was not considered appropriate; therefore, meta-analysis was not possible.

## Results

**Fig. 1.**
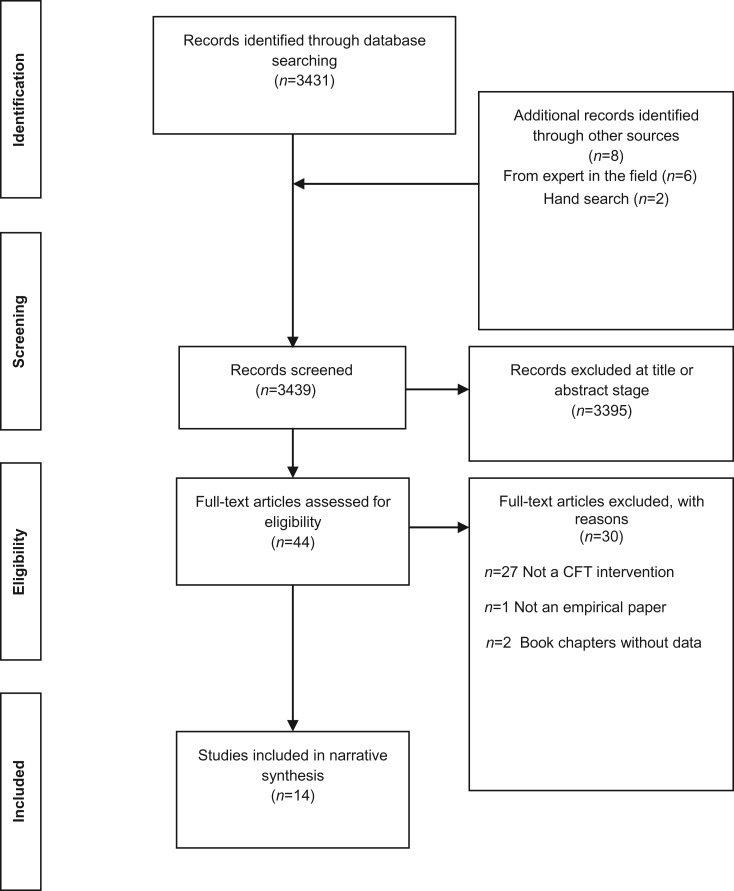
PRISMA (Preferred Reporting Items for Systematic Reviews and Meta-Analyses) flow diagram (Moher *et al*. 2009). CFT, Compassion-focused therapy.

Searching the electronic databases yielded 3431 records. Contact with experts in the field resulted in an additional six records, with two further records identified through bibliography scanning of key papers. Figure 1 shows the results of the search. Forty-four full-text articles were retrieved for consideration. Of these, 30 were subsequently excluded. Reasons for exclusion were: not a CFT intervention (*n* = 27); not an empirical paper (*n* = 1); and book chapters without data (*n* = 2). The remaining studies were included in the review (*n* = 14). A summary description of the key aspects of all studies is presented in Table 1.

The review searched for any interventions that hypothesized psychotherapeutic effects for interventions based on CFT or CMT. We did not exclude self-help interventions, and a distinction should be made between substantive CFT and isolated techniques, for example imagery generation delivered in a self-help format online. Studies ranged in the complexity of the intervention (see Table 1), and the duration and method of delivery. Table 2 describes the total duration and method of delivery of the interventions described. Ten studied clinical populations and four studied non-clinical samples. All the clinical samples received multi-component CFT delivered by a therapist whereas some non-clinical samples received only brief self-help interventions.

**Table 2.** Nature of CFT interventions

Study	Clinical/non-clinical population	Therapist details	Duration of intervention	CFT/CMT component
Braehler *et al*. 2013	Clinical	Two psychologists with experience using psychological therapy for psychosis. Five trial therapists (two consultants, three specialist psychologists) attended a 3-day workshop on attachment and interpersonal process in psychosis. Four of the five previously attended a 3-day workshop in CFT	32 h	CFT and TAU
Kelly *et al.* 2010	Non-clinical	Self-help using audio guide	n.r.	Brief CMT training but multi-component
Shapira & Mongrain, 2010	Non-clinical	Self-help using online resources	n.r.	Single self-compassion component
Beaumont *et al*. 2012	Clinical	A single qualified and BABCP-accredited cognitive behavioural psychotherapist	Minimum 12 h	CMT as part of substantive CBT
Kelly *et al.* 2009	Non-clinical	Self-help using slide show	Maximum 2 h	‘CFT’ but no therapist
Gilbert & Irons, 2004	Clinical	CFT trained clinical psychologist	6 h	Single component ‘critical thinking diaries’
Gilbert & Procter, 2006	Clinical	Therapist-led	24 h	Substantive multi-component CMT
Laithwaite *et al*. 2009	Clinical	Two chartered clinical psychologists, an advanced practitioner, a trainee clinical psychologist and two assistant psychologists. The group session was delivered by three therapists (for security reasons)	Minimum 20 h	Substantive multi-component CFT
Lucre & Corten, 2013	Clinical	The senior clinician is an accredited cognitive behavioural psychotherapist and both therapists had attended a 3-day CMT course. The co-therapist is a band four group facilitator. Both attended Paul Gilbert's CFT bimonthly supervision group for the duration of the 16-week group	n.r.	Substantive multi-component CFT
McEwan & Gilbert, unpublished data	Non-clinical	Self-help using sound recordings	Flexible: dependent on individual's choice. Minimum requirement was 70 min	Multi-component CMT
Judge *et al.* 2012	Clinical	Therapists who had attended a brief 3-day training course in CFT	24–28 h dependent on how many sessions participants attended	Substantive multi-component CFT
Gale *et al.* 2014	Clinical	All members of the team facilitating the recovery programme had received formal training and/or supervised practice in both CBT and CFT	Approximately 48–50 h	Substantive CBT integrating multi-component CFT
Mayhew & Gilbert, 2008	Clinical	One chartered clinical psychologist, specialized in working with adults with complex or severe mental health problems	12 h	Multi-component CFT
Ashworth *et al.* 2011	Clinical	One clinical psychologist	44	Multi-component CFT

CFT, Compassion-focused therapy; CMT, compassionate mind training; CBT, cognitive behavioural therapy; BABCP, British Association for Behavioural and Cognitive Psychotherapies; TAU, treatment as usual; n.r., not recorded.

### Quality assessment

A summary of the quality assessment for all included studies for core quality domains using a modified form of the Cochrane risk of bias tool (Higgins *et al.* 2011) is shown in Table 3. Of the total 14 studies retrieved through the searches, three were RCTs, two were non-RCTs, seven were observational studies, one was a case series and one was a case report (see Table 1).

**Table 3.** Summary of quality assessment for included studies

Study design	Studies	Potential areas of concern during quality assessment
RCTs	Kelly *et al.* 2010; Shapira & Mongrain, 2010; Braehler *et al*. 2013	High risk of attrition bias in two of the three studies due to high drop-out rate
Non-RCTs	Kelly *et al.* 2009; Beaumont *et al*. 2012	High risk of allocation bias due to group allocation following an initial assessment
Uncontrolled/observational studies	Gilbert & Irons, 2004; Gilbert & Procter, 2006; Mayhew & Gilbert, 2008; Laithwaite *et al*. 2009; Judge *et al.* 2012; Lucre & Corten, 2013; Gale *et al*. 2014; McEwan & Gilbert, unpublished data	High risk of allocation bias as all cases selected by condition with no control group
		High risk of performance bias and no blinding for researchers or participants
		Unclear risk of bias for attrition bias as incomplete outcome data in six out of eight studies
Case series	Ashworth *et al.* 2011	High risk of all bias categories due to selective reporting of one case

RCT, Randomized controlled trial.

### Summary of RCTs

Three RCTs were retrieved by the search (Kelly *et al*. 2010; Shapira & Mongrain, 2010; Braehler *et al*. 2013). Only one of these used a clinical sample. Braehler *et al*. (2013) studied a group of 40 individuals with a diagnosis of schizophrenia spectrum disorder with psychotic features whereas Kelly *et al*. (2010) studied a population of 119 smokers, and Shapira & Mongrain (2010) recruited a non-clinical sample of 1002 participants using the internet. Kelly *et al*. (2010) and Shapira & Mongrain (2010) both administered an experimental self-compassion intervention based on CMT. The experimental intervention in the Kelly study focused on imagery-based self-talk exercises designed to stimulate the self-soothing system, with two experimental control arms (self-energizing and self-controlling imagery) and one baseline control (daily self-monitoring). The experimental intervention in the Shapira & Mongrain (2010) study involved compassionate letter-writing, compared to one control condition where participants wrote freely about an early memory, and one experimental control condition where the letter-writing exercise focused on optimistic visualization. Reflecting the findings from the previous study types, the clinical study (Braehler *et al*. 2013) was more in-depth and had a longer duration than the two non-clinical studies [16 weekly 2-h sessions compared to 3 weeks of daily exercises (Kelly *et al*. 2010) or daily exercises over 7 days (Shapira & Mongrain, 2010)]. Braehler's experimental intervention comprised group CFT designed for psychosis, plus TAU. The control condition was TAU, which was free to vary. For primary outcomes of interest, Braehler *et al*. (2013) found CFT to decrease depression more that the control group whereas Shapira & Mongrain (2010) found both experimental conditions significantly decreased depression and improved happiness. Braehler *et al*. (2013) additionally found the CFT group displayed more compassion at the end of the study than TAU. Kelly *et al*. (2010) also found no difference between the two experimental control conditions for their main outcome of rate of smoking reduction, although both groups reduced smoking more than the baseline control.

### Summary of non-RCTs

Two of the retrieved studies had a control arm but had no formal randomization procedure (Kelly *et al*. 2009; Beaumont *et al*. 2012). Both of these studied clinical populations: Beaumont and co-workers studied 32 individuals requiring treatment after trauma and Kelly and colleagues studied 75 distressed chronic acne sufferers. The experimental arm of both studies received CFT/CMT, which involved compassionate letter-writing and generation of compassionate imagery with a focus on warmth, love, caring and acceptance. Participants in Beaumont's experimental group also received cognitive behavioural therapy (CBT) whereas the control arm received CBT only. The Kelly study reported two control arms, one arm receiving no exercises and the other receiving an attack-resisting intervention that focused on strength, self-confidence, resistance and resilient imagery. Neither the Kelly study nor the Beaumont study reported effect sizes. Kelly *et al*. (2010) reported a small to medium effect size according to Cohen's criteria (1988). This study was shorter in duration at 2 weeks whereas the Beaumont study consisted of 12 weekly sessions. Both studies reported that depression had significantly improved by the end of the study period across all conditions. Beaumont *et al*. (2012) reported that the improvement was greater for the CMT condition than for the control whereas Kelly *et al*. (2009) found that only the attack-resisting condition lowered depression more than the ‘no exercise’ control. Beaumont *et al*. (2012) found no difference in anxiety levels between the two study groups. There was a greater improvement in self-compassion for the CMT group over the control group. None of the included RCTs reported effect sizes.

### Summary of case and observational studies

The case series and case study included in the review focused on individuals who had been clinically diagnosed with psychological disorders. Mayhew & Gilbert (2008) studied the effect of CMT on the psychological symptoms of three patients diagnosed with schizophrenia who were experiencing auditory hallucinations. Ashworth *et al.* (2011) report on the effects of CFT combined with CBT on a single female with acquired brain injury following a road traffic accident. Although these studies included a range of baseline and outcome measurements (e.g. inadequate-self, fear of self-compassion, depression, anxiety), they both also targeted specific outcomes relevant to their clinical populations. All three participants in the Mayhew & Gilbert (2008) study experienced less malevolent and less persecuting voices after 12 weeks of CMT. Two of the three heard more reassuring voices. After 24 weeks of CFT with CBT, the participant in the study by Ashworth *et al.* (2011) demonstrated a reduction in anger expression to within a normal range. Beliefs relating to key cognitions relating to an existing co-morbid eating disorder indicated improvement.

Seven observational studies with no control arm were identified through the search. Six out of seven of these studied clinical populations. Gilbert & Irons (2004) focused on eight individuals from a self-help depression group who regarded themselves as self-critical, Gilbert & Procter (2006) studied six patients being treated for major to severe long-term and complex mental health issues, Laithwaite *et al*. (2009) focused on 18 individuals with psychosis, and Lucre & Corten (2013) studied 10 individuals with chronic personality disorder who regarded themselves as self-critical. One of the observational studies used a non-clinical sample, studying the effects of compassionate imagery in 45 volunteers (McEwan & Gilbert, unpublished data). Judge *et al*. (2012) studied the effects of group CFT on a population with a range of diagnoses, including depression, anxiety and bipolar disorder. Gale *et al*. (2014) studied group-based CBT, integrating CFT on a population of individuals diagnosed with an eating disorder. Target outcomes for these studies tended to focus on general measures of elements of psychological well-being, such as depression (all seven studies), anxiety (Gilbert & Irons, 2004; Gilbert & Procter, 2006; Judge *et al.* 2012; McEwan & Gilbert, unpublished data) and eating behaviour (Gale *et al.* 2014); and also targeted outcomes specifically relevant to the aims of CFT, that is reduction in self-criticism and increased self-compassion (Gilbert & Irons, 2004; Gilbert & Procter, 2006; Laithwaite *et al*. 2009; Judge *et al*. 2012; Lucre & Corten, 2013; McEwan & Gilbert, unpublished data).

CFT sessions in the studies of clinical populations were generally lengthier and more in-depth than those using non-clinical populations, ranging in duration from four 1-h sessions over 7 weeks (Gilbert & Irons, 2004) to 20 sessions over 10 weeks (Laithwaite *et al*. 2009), 24 sessions over 20 weeks (Gale *et al*. 2014) and 16 weeks of therapy followed by a 1-year follow-up (Lucre & Corten, 2013). The McEwan & Gilbert study of a non-clinical sample entailed daily self-practice of compassionate imagery over a 2-week period. In terms of primary outcomes of interest, five of the observational studies showed a reduction in depression (Gilbert & Procter, 2006; Laithwaite *et al*. 2009; Judge *et al*. 2012; Lucre & Corten, 2013; McEwan & Gilbert, unpublished data) and three demonstrated a reduction in anxiety (Gilbert & Procter, 2006; Judge *et al.* 2012; McEwan & Gilbert, unpublished data). Four of the studies observed a reduction in self-criticism (Gilbert & Procter, 2006; Laithwaite *et al*. 2009; Judge *et al.* 2012; McEwan & Gilbert, unpublished data) and five reported an increase in self-compassion (Gilbert & Irons, 2004; Gilbert & Procter, 2006; Beaumont *et al*. 2012; Judge *et al*. 2012; Braehler *et al*. 2013).

### Moderators

Three of the studies reported that effects of the experimental interventions were moderated by personality factors. Kelly *et al.* (2010) found the self-compassion intervention reduced smoking at a quicker rate for: those low in readiness-to-change; those high in self-criticism; and those with more vivid imagery. Shapira & Mongrain (2010) reported moderating effects for self-criticism and dependence, and Kelly *et al.* (2009) reported moderating effects for trait self-criticism, with those higher in trait self-criticism in the attack-resisting condition reporting lower depression at time 2 than those low in trait self-criticism. McEwan & Gilbert (unpublished data) showed that those higher in baseline scores of avoidance attachments and those scoring higher in inadequate self at baseline were associated with larger reductions in depression. Additionally, individuals with higher scores on inadequate self-criticism showed higher scores on trying to resist compassionate emotions and feeling tense.

### Tolerance and acceptability

#### Attrition/drop-outs

Attrition/drop-outs ranged from low to high. In the study by Braehler *et al*. (2013), four of the 40 participants dropped out overall, all of whom were from the CFT condition (completers: 90%). It should be noted, however, that the control group for this study was TAU, and these patients would be expected to be followed up as a routine part of their care. Therefore, drop-outs from this group would have to be active avoiders. Judge *et al*. (2012) reported that 27/42 (completers: 64%) completed the follow-up questionnaire, with six out of 42 clients attending less than eight sessions. Gale *et al*. (2014) reported attrition of 38/139 clients who did not complete the programme (completers: 73%). Kelly *et al*. (2010) reported that 24/126 dropped out before completion of the study (completers: 81%), although χ^2^ tests showed these were evenly distributed between groups. Shapira & Mongrain (2010) reported an overall drop-out of 799/1002 (completers: 20%). Those who adhered to the entire programme were statistically less needy, less depressed and older at baseline. Drop-outs were low in the remaining studies. Reasons for attrition were not always given. Reasons included participants feeling better, becoming physically unwell or feeling too upset in sessions (Gilbert & Procter, 2006).

#### Compliance

Kelly *et al*. (2010) reported no statistical difference in compliance rates between conditions. Gilbert & Irons (2004) reported that all participants had experienced problems keeping their self-criticism diaries over the study period.

#### Tolerance and acceptability of CFT

Experiences of generating compassionate images were recorded in some studies. Gilbert & Irons (2004) reported one participant finding her compassionate other turning into a figure who reminded her of her ex-husband, which made the experience unpleasant. Another participant reported easily imagining warmth but finding it less easy to imagine acceptance. Other participants reported difficulties holding onto their compassionate image for more than a fleeting time. Lucre & Corten (2013) present qualitative data relating to their participants’ reflections of CFT. Fear of compassion was a recurring theme. Many participants associated warmth and kindness with self-indulgent/self-destructive behaviour or inactivity. McEwan & Gilbert (unpublished data) describe participants reporting difficulties generating compassionate images or finding time to practise. Others report mixed compassionate feelings with other emotions such as pity and sadness. Those high in self-criticism responded more negatively to CFT than those low in self-criticism. Mayhew & Gilbert (2008) report one participant's struggle to develop self-compassion. His compassionate image was self-critical and condemning of him rather than compassionate, and therefore intervention was needed to generate an alternative. Ashworth *et al.* (2011) describe their participant as reacting positively to the process of CFT.

## Discussion

The aim of the present study was to provide an early review of the research evidence regarding the effectiveness of CFT as a psychotherapeutic intervention, and to provide information on its acceptability and tolerability that may be used to inform future trials. The empirical studies retrieved for this review build on a body of correlational evidence indicating that compassion may be a promising target upon which to focus psychotherapeutic intervention, particularly for individuals who are high in self-criticism. The small number of controlled studies that were retrieved in this review highlights the novelty of CFT, although the number of recently published, in press and unpublished studies suggests that this is a growing focus for psychotherapeutic intervention. The current review specifically searched for and evaluated studies of CFT; however, the body of research identified is a reflection of a wider movement towards the integration of ‘compassion’ in promoting psychological well-being outside of the clinical population (e.g. Hofmann *et al.* 2011; Jazaieri *et al.* 2013; Singer & Bolz, 2014). The review also highlights an increasing application of this therapy to address psychological disorders beyond depression, including schizophrenia and psychosis.

### Quality of evidence

One aim of the current review was to provide recommendations for future trials of CFT to ensure that these trials would satisfy the requirements for evidence-based practice. The relevance of EBM to clinical psychology is increasingly recognized (Spring & Neville, 2011). Of particular importance to EBM is the quality of the available evidence. RCTs offer a potentially reliable method of assessing the effectiveness of psychotherapeutic interventions. The National Institute for Clinical Excellence (NICE) use an established evidence hierarchy when evaluating evidence on clinical effectiveness (Atkins *et al.* 2004; NICE, 2004). This is to ensure not only that new treatments are safe and effective but also that their introduction into the health system does not displace existing treatments that are equally, or more, efficacious, safe and cost-effective. RCTs are at the top of this hierarchy, and it is widely accepted that an RCT design offers the highest internal validity, is effective at minimizing bias, and is the recommended study design for clinical effectiveness evidence (Nutbeam, 1998; Hawe *et al*. 2004).

However, there is some debate regarding the appropriateness of RCTs for evaluation of some psychological therapies. Gilroy (2006) claims that RCTs do not adequately address how or why a psychological intervention is effective, and that such trials fail to take into account the complexity of an individual. Wood *et al.* (2011) recommend the use of mixed methods research designs with long-term follow-up. Issues around blinding and variation in the practices of individual therapists also result in difficulties when conducting RCTs for psychotherapies. However, notwithstanding the complexities of both intervention and therapist, we would argue that a rigorously designed and well-conducted RCT is the most appropriate method of providing a fair test of any new treatment.

Case and observational studies identified in this review indicate that CFT can significantly improve psychological well-being, and therefore seems to be more effective than no treatment. However, there is still insufficient high-quality evidence to demonstrate that CFT is more effective than current standard treatments, for example CBT or other imagery-based interventions. Future studies should investigate whether CFT can be a rival treatment to CBT or counselling or whether it should be considered as a concomitant therapy. Indeed, CFT involves teaching skills generic to conventional therapy such as Socratic dialogues, inference chaining, thought and emotion monitoring, behavioural experiments exposure, and use of imagery.

One study identified in the review (McEwan & Gilbert, unpublished data) includes a 6-month follow-up that shows that the positive effects of CFT are maintained in the longer term with the exception of anxiety; however, most other studies only report short-term outcomes. Well-designed larger-scale trials with adequate follow-up should aim to establish where CFT might fit into the care pathway for people undergoing psychotherapy, and attempt to disentangle the effectiveness of individual components of such complex interventions. Evaluation of the existing trials raised several issues of interest, as follows.

### Understanding and quantification of compassion

The success of using compassion in psychotherapeutic intervention is dependent on recipients fully understanding the nature and meaning of compassion. Qualitative and anecdotal data from the studies included in the current review highlighted several incidents where participants had misunderstood ‘compassion’ (Gilbert & Irons, 2004; Mayhew & Gilbert, 2008; Lucre & Corten, 2013; McEwan & Gilbert, unpublished data; McEwan *et al.,* unpublished data). This may be particularly pertinent for experimenters planning larger-scale trials; the low number of participants included in the clinical studies meant that individuals could be guided and monitored through the process of generating self-compassionate imagery. Although imagery experience was recorded and evaluated in two studies (Gilbert & Procter, 2006; McEwan & Gilbert, unpublished data), none of the studies examined here reported formal manipulation checks to ensure that ‘compassion’ was being generated as intended.

The observational and case reports included in this review showed positive results for participants after CFT. In particular, reductions in depression were observed in the majority of studies. However, for studies with control arms, improvements in target outcomes were often observed in both experimental and control conditions (Kelly *et al*. 2009, 2010; Shapira & Mongrain, 2010; Beaumont *et al*. 2012). The studies using clinical populations understandably developed more complex and in-depth interventions than studies using non-clinical samples. Despite the correlational evidence to suggest that increases in compassion are related to improved psychological well-being, none of the studies included in this review specifically analysed whether ‘compassion’ mediated the relationship between intervention and outcome. Previous compassion research has attempted to analyse the specific contribution of compassion to the psychotherapeutic process. Neff & McGehee (2010) explored the mediating role of self-compassion. They found self-compassion partially mediated the relationship between maternal support, family functioning and attachment style and well-being. Kuyken *et al.* (2010) demonstrated that the effect of mindfulness-based cognitive therapy (MBCT) on depressive symptoms was mediated by self-compassion. Future trials are needed to identify whether compassion is the ‘active’ component responsible for improved outcomes, and what additional benefit ‘compassion’ adds to conventional therapies such as CBT or counselling. Further study exploring the specific components of CFT may also help to identify the factors that distinguish CFT from simple compassion exercises. In Beaumont's (2012) non-RCT, both ‘CFT plus CBT’ and ‘CBT alone’ conditions showed an improvement in depression and avoidance symptoms, although the ‘CFT plus CBT’ group showed greater improvement than ‘CBT alone’. CFT was also used alongside CBT in the Gale *et al*. (2014) study, and indeed proponents of CFT argue that techniques promoting compassion will enable clients to better engage with their current therapies [e.g. CBT, exposure and response prevention (ERP)]. Studies included in this review varied in the duration and delivery method of the interventions. They also comprised both single and multiple components. The importance of each of these factors therefore needs further study. Jazaieri *et al.* (2013) show how practice effects (i.e. the amount of formal compassion meditation practice) influence levels of compassion for self and others, with more practice associated with greater levels of compassion. The benefits of single-component, short-duration interventions may be limited.

### Specificity of effects of CFT

The studies identified in this review indicated some evidence for specificity of effects, particularly the finding that the effect of CFT can be moderated by self-criticism (Kelly *et al*. 2009, 2010; Shapira & Mongrain, 2010) and other individual differences, with highly self-critical individuals showing greater improvement in symptoms than those low in self-criticism (Kelly *et al*. 2010; Shapira & Mongrain, 2010). This suggests that CFT may be a promising tailored intervention for specific individuals. McEwan & Gilbert (unpublished data) showed that those high in self-criticism gave more negative feedback and showed initial resistance to CFT than those low in self-criticism. Other studies have noted initial resistance and difficulties with CFT for those high in self-criticism (Mayhew & Gilbert, 2008). However, data from the McEwan & Gilbert study showed that those who were high in self-criticism who practised overcame their initial resistance and showed improvement. Further studies have also shown that it is possible to overcome initial resistance, and that even individuals showing resistance or fear of compassion begin to become more affiliative to themselves and others after prolonged training (Jazaieri *et al.* 2013).

### Acceptability of CFT

The studies identified in this review offer some insight into the acceptability of CFT to study participants. Studies of non-clinical populations saw large attrition (Kelly *et al*. 2010; Shapira & Mongrain, 2010) but CFT in the clinical context seemed to be acceptable to participants as part of a treatment regime. The findings relating to acceptability of CFT from the studies in this review support qualitative work by Pauley & McPherson (2010). In this study, participants understood compassion to be ‘kindness and action’, and felt it would be useful, but they also felt that being self-compassionate would be difficult and challenging as a result of their psychological disorders negatively impacting on their ability to be self-compassionate. McEwan & Gilbert (unpublished data) noted large variation in the time spent practising compassionate imagery when participants were given freedom to choose. This adds to findings from a study by Rockliff *et al*. (2008), where a dataset had to be dropped from analyses because participants were complaining of boredom and fatigue during their second 5-min session of compassion imagery. Both of these studies were of a non-clinical sample, however, and it may be that CFT is more acceptable to individuals in a clinical setting where some form of psychotherapy is expected and desired.

### Implementation fidelity

Larger-scale clinical trials are needed and they will need to address issues surrounding implementation fidelity, that is the degree to which an intervention or programme is delivered as intended. Where therapist experience and training were reported in the studies in this review, variation in the level of CFT training that had been undergone was found, and it is unclear in many studies the level to which expert supervision was provided. This results in uncertainty around the fidelity of the interventions provided. More uniform structure of therapy is difficult in a clinical setting; however, a high-quality large-scale trial will require multiple therapists across multiple sites, which may introduce variation that could affect the credibility of the research. Carroll *et al.* (2007) developed a framework within which implementation fidelity can be measured, and the use of such a framework in a large clinical trial may protect against the threat of variation.

## Conclusions

There is increasing interest in CFT as a psychotherapeutic intervention. CFT is novel in that it focuses on the evolution of human affiliative behaviour. CFT is proposed as a multi-modal therapy, incorporating aspects of other evidence-based therapies. Therapists from a range of disciplines can use knowledge and understanding of the biological basis of the affect regulation systems, and how these may be affected by early development as a framework within which to deliver effective psychotherapy. This early systematic review of the current evidence suggests that CFT may be more effective than no treatment or as effective as TAU in treating psychological disorders. Specifically, CFT shows promise as an intervention for individuals high in self-criticism. However, the evidence is currently insufficient to show that CFT is more effective when compared to current standard treatments such as CBT or other imagery-based interventions. This conclusion is based on a lack of large-scale, high-quality trials, and is not an indication of the existence of negative evidence. Future trials are therefore needed that retain intervention fidelity while including a design that is appropriate to the evaluation of complex, individually tailored interventions.
